# Effects of Maternal Pre-Pregnancy BMI and Gestational Weight Gain on the Development of Preeclampsia and Its Phenotypes: A Prospective Cohort Study in China

**DOI:** 10.3390/jcm11195521

**Published:** 2022-09-21

**Authors:** Senmao Zhang, Xing Qiu, Jiabi Qin, Xingli Song, Yiping Liu, Jianhui Wei, Mengting Sun, Jing Shu, Tingting Wang, Lizhang Chen, Yurong Jiang

**Affiliations:** 1 Department of Epidemiology and Health Statistics, Xiangya School of Public Health, Central South University, Changsha 410078, China; 2 Hunan Provincial Key Laboratory of Clinical Epidemiology, Changsha 410078, China; 3 Xiangya School of Nursing, Central South University, Changsha 410013, China; 4 National Health Commission Key Laboratory for Birth Defect Research and Prevention, Hunan Provincial Maternal and Child Health Care Hospital, Changsha 410028, China; 5 Department of Obstetrics, Hunan Provincial Maternal and Child Health Care Hospital, Changsha 410028, China

**Keywords:** pre-pregnancy BMI, gestational weight gain, preeclampsia, China, cohort

## Abstract

Preeclampsia (PE) is a common and serious pregnancy-specific disorder, which is closely linked with adverse maternal and neonatal outcomes. This study aimed to evaluate whether maternal pre-pregnancy body mass index (BMI) and gestational weight gain (GWG) was associated with preeclampsia and its phenotypes. In this prospective study, 32,531 women with singleton pregnancies were finally included. Compared with women with normal pre-pregnancy BMI, women with overweight and obesity were at increased risk of PE (RR = 1.62, 95%CI: 1.57–1.66; RR = 2.04, 95%CI: 1.97–2.11, respectively), while those who were underweight had a lower risk of PE (RR = 0.84, 95%CI: 0.81–0.88). When compared with women who gained adequate GWG, pregnant women with inadequate GWG and excessive GWG had an increased risk of PE (RR = 1.15, 95%CI: 1.12–1.19; RR = 1.56, 95%CI: 1.52–1.60, respectively). The observed increased risk was generally similar for mild-, severe-, early- and late-onset PE, and the reduced risk was similar for severe- and late-onset PE. No significant interactions between GWG and pre-pregnancy BMI on the risk of PE were identified (*p*-interaction > 0.05). In conclusion, pre-pregnancy overweight or obesity and excessive GWG have established risk factors for PE, and that the potential risk may vary according to PE phenotypes. Moreover, the synergistic effect that may exist between pre-pregnancy BMI and GWG.

## 1. Introduction

Preeclampsia (PE) is a common and serious pregnancy-specific disorder characterized by maternal new-onset hypertension and proteinuria after 20 weeks of gestation, which represents a great threat to maternal and neonatal health [1]. The prevalence of PE has been rising in recent years, with about 5–8% worldwide, becoming a major health concern around the world [2,3]. PE is the major cause of maternal and neonatal mortality [4], and it has also been reported to be associated with intrauterine growth restriction [5], prematurity [6], pregnancy-related intensive care unit admissions [7], and cardiovascular disease later in life [8,9]. Given the known and potential adverse consequences of PE to both the mother and offspring, identifying the underlying risk factors for PE is crucial for the implementation of preventive actions.

Recent evidence suggests that pre-pregnancy body mass index (BMI) and gestational weight gain (GWG) are independent and modifiable factors for the adverse complications of pregnancy [10,11]. Being overweight and obese among women is the major clinical and public problem in obstetrics that affects both the mother and the fetus [12]. The World Health Organization (WHO) estimates that the prevalence of overweight and obesity among women has increased dramatically in the last 40 years [13]. Currently, reaching 40% of women are overweight while 15% were obese worldwide [13]. GWG is a normal part of a healthy pregnancy and is necessary for healthy fetal development [14], but excessive GWG can contribute to adverse pregnancy outcomes [15,16,17,18]. Both pre-pregnancy obesity and excessive GWG are connected with PE through increased oxidative stress, stimulating a systemic inflammatory response of vessels, and ultimately accelerating vascular endothelial cell damage [19,20]. Findings from different countries and study populations have reported that pre-pregnancy overweight and obesity are connected with a higher risk of PE [15,16,20,21,22,23,24,25,26]. In addition to pre-pregnancy BMI, previous studies have examined the association between GWG and PE in recent years [27,28,29,30,31,32,33]. Several studies suggested that excessive GWG is associated with an increased risk of PE [9,27,28,29]. However, the association between GWG and the risk of PE remains controversial, with some studies demonstrating no correlations [30,31,32,33] due to differences in study populations, small study sample sizes, or differences in classification criteria. Furthermore, although previous studies have investigated the association between pre-pregnancy BMI, GWG and the risk of PE [23,24,25], no study has explored the association of pre-pregnancy BMI and GWG with PE and different phenotypes based on a large-sample, prospective cohort design.

Considering the persistently rising prevalence of PE in populations and its potential effect on maternal and neonatal health, as well as inconsistent results of existing literature, it is of great importance to understand more about the association between pre-pregnancy BMI, GWG and risk of PE. Therefore, we did a prospective cohort study of almost 32,531 pregnant women in central China to explore the association of pre-pregnancy BMI and GWG with PE and its various phenotypes to provide a theoretical basis for PE prevention.

## 2. Materials and Methods

### 2.1. Study Design and Subjects

This study was nested on a prospective cohort study that was carried out at Hunan Provincial Maternal and Child Health Care Hospital, a provincial care center for mothers and children in Hunan Province, Central China. Our study complied with the principles of the Declaration of Helsinki. All pregnant women signed informed consent before data collection, and ethics approval was obtained from the Ethics Committee of Xiangya School of Public Health Central South University (No. 84 XYGW-2018-36). Moreover, this prospective study has been registered in the Chinese Clinical Trial Registry center (registration number: ChiCTR1800016635).

From 13 March 2013 to 31 December 2020, pregnant women were invited if they were ≥18 years old, at 8–14 weeks of gestation, planned to receive their antenatal care at our study hospital during the entire pregnancy, and provided informed consent for participation. However, pregnant women were excluded if they were multiple pregnancies, artificial fertilization, termination of pregnancy, chronic hypertension diagnosed prior to pregnancy, or gestational hypertensive disorders except for PE. After being recruited, pregnant women who consent to participate were interviewed face-to-face by a trained researcher using a self-designed, structured questionnaire to collect information on maternal characteristics and pre-pregnancy BMI. Detailed information on maternal GWG, PE, and infant characteristics was gathered from medical records.

### 2.2. Outcome Definition

PE was diagnosed according to guidelines for diagnosis and treatment of hypertensive diseases in pregnancy (2015) developed by the Hypertensive Diseases in Pregnancy Group of the Obstetrics and Gynecology Branch of the Chinese Medical Association [34], which was defined as maternal new-onset hypertension (systolic blood pressure ≥ 140 mmHg and/or diastolic blood pressure ≥ 90 mmHg) after 20 weeks of gestation accompanied any of the following systemic complications: the urine protein ≥ 0.3 g/24 h, the urinary protein-to-creatinine ratio ≥ 0.3, the random urine protein ≥(+) (when urinary protein quantization is not available), no proteinuria but with involvement of heart, lung, liver, kidney, blood system, digestive system, nervous system or placenta-fetus, etc. PE can be further subclassified as early-onset PE (EOPE) (<34 weeks of gestation) and late-onset PE (LOPE) (≥34 weeks of gestation), as well as severe PE (SPE) and mild PE (MPE) [35]. SPE was diagnosed as proteinuria and consistently elevated blood pressure (≥160/110 mmHg) with other additional serious symptoms (e.g., persistent headache, visual disorders, constant epigastric pain, reduced urine volume, heart failure, fetal growth restriction, etc.). MPE was diagnosed as proteinuria and elevated blood pressure (140–160/90–110 mmHg), but without additional serious symptoms.

### 2.3. Exposure Definition

The prevalence of obesity in China and the rest of the world is increasing in recent decades, including women of childbearing age [36]. The recommended BMI cut-off points for determining overweight and obesity were controversial, as the percentage of body fat for a given BMI is higher in Asian populations than in Caucasians of similar age and sex [37,38]. As such, the BMI cutoffs for overweight and obesity differ between Asian and Western populations, and the BMI criterion for Asian populations should be lowered to better fit the characteristics of this racial group. In our study, the standards of BMI cut-off points were based on the Working Group on Obesity in China (WGOC) and the International Life Sciences Institute Focal Point in China, which was categorized as underweight (˂18.5 kg/m^2^), normal weight (18.5–23.9 kg/m^2^), overweight (24.0–27.9 kg/m^2^), and obesity (≥28.0 kg/m^2^) [39].

In addition, since there were no official recommendations standards for GWG in the Chinese population, the 2009 Institute of Medicine (IOM) GWG guideline was used in our study [14]. The range of recommended GWG for underweight pregnant women (BMI < 18.5), normal-weight pregnant women (BMI = 18.5–23.9), overweight pregnant women (BMI = 24.0–27.9), and obese pregnant women (BMI ≥ 28.0) are 12.5–18.0 kg, 11.5–16.0 kg, 7.0–11.5 kg, and 5.0–9.0 kg, respectively. GWG was then classified into inadequate GWG (below the recommended range), adequate GWG (within the recommended range), and excessive GWG (above the recommended range).

### 2.4. Statistical Methods

Descriptive statistics were used to assess the baseline characteristics of study participants, and the Chi-square test was employed to compare categorical variables. To further control for potential selection bias, inverse probability weighting (IPW) was estimated using the calculated propensity scores from multivariable logistic regression. This model included the following variables: maternal age, education level, annual income, residence, parity, family history of hypertension, active smoking in early pregnancy, passive smoking in early pregnancy, drinking in early pregnancy, and gestational diabetes mellitus. The variables chosen were based on a literature review, clinical experience, and theoretical considerations. The PE group was weighted by 1/PS, whereas the non-PE group was weighted by 1/(1 − PS). For each baseline characteristic, the standardized mean difference (SMD) between the two groups was calculated before and after IPW to assess whether the IPW was adequately controlled for potential bias. SMD less than 10% indicates a relatively small degree of imbalance [40]. The associations of pre-pregnancy BMI, total GWG with the risk of PE and its phenotypes were assessed by relative risk (RR) and their corresponding 95% confidence interval (CI), computed with modified Poisson regression. All data used EpiData software, version 3.1 (EpiData Association, Odense, Denmark) for double entry and validation. Data cleaning and data analysis were performed with R software, version 3.5.0 (R Foundation for Statistical Computing, Vienna, Austria). All tests were two-sided and the significance level was set at 0.05.

## 3. Results

### 3.1. Characteristics of Participants

A total of 32,531 women with singleton pregnancies were included in the present analysis of which 788 pregnant women were diagnosed with PE. Among those women with PE, 356 were diagnosed with M-PE and 432 with S-PE, while 331 were with EOPE and 457 were with LOPE. The incidence rate of PE, M-PE, S-PE, EOPE, and LOPE was 2.37% (95%CI: 2.21–2.54%), 1.30% (95%CI: 1.18–1.43), 1.07% (95%CI: 0.97–1.19), 1.00% (95%CI: 0.89–1.10), and 1.37% (95%CI: 1.25–1.50), respectively.

The baseline characteristics of the unweighted and IPW-weighted cohorts are summarized in Table 1. In the unweighted cohort, there were statistically significant differences among the comparative groups across most covariates including maternal age, education level, annual income, residence, family history of hypertension, drinking in early pregnancy, and gestational diabetes mellitus (all *p* < 0.05). The propensity scores showed considerable overlap between the comparative groups in these spreads, which indicated that excellent covariate balance can be achieved with weights. After IPW, all the covariates were well balanced between the two groups (SMD < 10% for all variables).

### 3.2. Association of Pre-Pregnancy BMI and Total GWG with the Risk of PE

Table 2 shows the association of maternal pre-pregnancy BMI and total GWG with the risk of PE. The multivariable-adjusted model (model 2) indicated that maternal pre-pregnancy overweight and obesity were significantly associated with an increased risk of PE compared with women with normal weight, while those who were underweight were not statistically linked to the risk of PE. When compared with women who gained adequate GWG, pregnant women with inadequate GWG and excessive GWG were also significantly associated with the development of PE. Because the baseline characteristics were imbalanced, we further used the IPW approach to assess the associated risks. In adjusted IPW models (model 3), we found that pre-pregnancy overweight (RR = 1.62, 95%CI: 1.57–1.66) and obesity (RR = 2.04, 95%CI: 1.97–2.11) were significantly correlated with an increased risk of PE compared with those with normal weight, while underweight (RR = 0.84, 95%CI: 0.81–0.88) was related to a reduced risk of PE. When compared to women who had adequate GWG, pregnant women with inadequate GWG (RR = 1.15, 95%CI: 1.12–1.19) and excessive GWG (RR = 1.56, 95%CI: 1.52–1.60) had a higher risk of PE.

### 3.3. Association of Pre-Pregnancy BMI and Total GWG with the Risk of PE Phenotypes

The association of maternal pre-pregnancy BMI and total GWG with PE phenotypes is summarized in Table 3. In a multivariable-adjusted model (model 2), our current study showed that maternal pre-pregnancy overweight and obesity had a significantly increased risk of MPE, SPE, EOPE, and LOPE compared with those with normal weight, whereas underweight was not statistically associated with the development of all PE phenotypes. In addition, our results indicated that mothers with inadequate GWG and excessive GWG had a significantly higher risk of most PE phenotypes compared to those with adequate GWG. In adjusted IPW models (model 3), compared with those with normal weight, we found that pre-pregnancy overweight and obesity were significantly associated with an increased risk of MPE (RR = 1.88, 95%CI: 1.81–1.96, RR = 2.93, 95%CI:2.78–3.07, respectively), SPE (RR = 1.85, 95%CI: 1.79–1.92, RR = 2.67, 95%CI: 2.56–2.79, respectively), EOPE (RR = 2.37, 95%CI: 2.28–2.46, RR = 3.06, 95%CI: 2.90–3.24, respectively), and LOPE (RR = 1.62, 95%CI: 1.56–1.68, RR = 2.53, 95%CI: 2.43–2.63, respectively). However, maternal underweight was relevant for a reduced risk of MPE (RR = 0.81, 95%CI: 0.76–0.86), SPE (RR = 0.82, 95%CI: 0.78–0.87), and LOPE (RR = 0.72, 95%CI: 0.68–0.76) compared with those with normal weight. Additionally, our results indicated that mothers with excessive GWG had a higher risk of MPE (RR = 1.51, 95%CI: 1.45–1.57), SPE (RR = 2.12, 95%CI: 2.04–2.19), EOPE (RR = 2.13, 95%CI: 2.04–2.21), and LOPE (RR = 1.64, 95%CI: 1.58–1.69) compared to women with adequate GWG. Meanwhile, our study also showed that mothers with inadequate GWG had a significantly increased risk of SPE (RR = 1.48, 95%CI: 1.42–1.54), EOPE (RR = 1.39, 95%CI: 1.33–1.46), and LOPE (RR = 1.09, 95%CI: 1.05–1.14).

### 3.4. Association of GWG with the Risk of PE and Its Phenotypes across Different Pre-Pregnancy BMI Status

The association of maternal inadequate GWG and excessive GWG with the risk of PE and its phenotypes across strata of different pre-pregnancy BMI levels are summarized in Table 4. Significant associations between inadequate GWG and excessive GWG and the risk of PE and its phenotypes were not only found in mothers with underweight but also in mothers with normal weight. Stratified analysis suggested that pre-pregnancy overweight/obese group had a higher incidence rate of PE compared with pre-pregnancy normal weight women or pre-pregnancy underweight women, regardless of inadequate or excessive weight gain during pregnancy. Additionally, excessive GWG or inadequate GWG was found to be a stronger risk factor for PE and different PE phenotypes in pre-pregnancy underweight than in women with normal weight or overweight/obesity. Nonetheless, no significant interactions between GWG and pre-pregnancy BMI were identified (*p* interaction > 0.05).

## 4. Discussion

### 4.1. Main Findings

In this large population-based cohort, the incidence rate of PE was about 2.37%, which was slightly lower than previous meta-analysis reported by our team (about 3.60%) [41]. Our results found that women with pre-pregnancy overweight or obesity were significantly correlated with an increased risk of PE compared with those with normal weight, while those who were underweight had a lower risk of PE in the IPW model. Previous studies from different countries and populations also reported that pre-pregnancy overweight and obesity are related to a higher risk of PE [15,16,20,21,22,23,24,25,26], which is consistent with our findings. However, pre-pregnancy underweight was not significantly associated with the risk of PE in previously published studies [20,21,22,23,24,25,26], which is similar to our results in the multivariable-adjusted model but is not consistent with our results in the IPW model. The discrepancy in the results may be due to the effect of potential bias and the different statistical methodologies. In addition, the present study suggested that pre-pregnancy overweight or obesity was also significantly relevant for an increased risk of PE phenotypes including MPE, SPE, EOPE, and LOPE compared with those with pre-pregnancy normal weight, whereas pre-pregnancy underweight was related to the reduced risk of MPE, SPE, and LOPE.

Additionally, our study found that pregnant women with inadequate GWG and excessive GWG had a significantly higher risk of PE and most PE phenotypes including SPE, EOPE, and LOPE when compared with those with adequate GWG, but not MPE. Existed studies also suggested that women with excessive GWG were significantly associated with the development of PE (reference no: [9,27,28,29]), which is consistent with our findings. This finding between inadequate GWG and an increased risk of PE was unexpected, as it was inconsistent with previous studies (reference No: [30,31,32,33]). The heterogeneity in these studies could be attributed to the differences in different geographical regions, study designs, or classification criteria. For example, populations in different geographic regions may have genetic differences and thus may contribute to the inconsistency of the study results. Otherwise, the classification criteria of GWG were inconsistent, and two different criteria (2009 IOM GWG Guidelines [9,27,28,29,30,31] and 1990 IOM GWG Guidelines [32] were adopted to classify GWG based on pre-pregnancy BMI in previous studies.

Meanwhile, a significant association between inadequate GWG and excessive GWG and the risk of PE and its phenotypes were not only observed in pregnant women with underweight but also existed in those with normal weight. Furthermore, pre-pregnancy overweight/obese group had a higher incidence rate of PE compared with pre-pregnancy normal-weight women or pre-pregnancy underweight women, regardless of inadequate or excessive weight gain during pregnancy. Additionally, excessive GWG or inadequate GWG was found to be a stronger risk factor for PE and different PE phenotypes in pre-pregnancy underweight than in women with normal weight or overweight/obesity. However, the synergistic effect between pre-pregnancy BMI and GWG was also not significant, which also warrants further elucidation in future studies.

### 4.2. Potential Mechanisms

Currently, the potential mechanisms involved in the association of maternal pre-pregnancy BMI and GWG with the risk of PE and its phenotypes remain uncertain. Previous research suggests that oxidative stress and systemic inflammatory response were an important part of PE pathogenesis [42,43]. Nowadays, overweight and obesity have been viewed as chronic inflammatory conditions that might trigger inflammation and oxidative stress, resulting in increased levels of certain inflammatory cytokines and C-reactive protein [44,45,46]. These inflammatory cytokines and proteinases could stimulate a systemic inflammatory response of vessels and accelerate vascular endothelial cell damage, ultimately leading to the clinical symptoms of PE [47,48]. In addition, our study found that women with excessive GWG had a significantly higher risk of PE and most PE phenotypes in pre-pregnancy underweight than in women with normal weight or overweight/obesity. Previous studies indicated that excessive GWG may lead to an increased risk of impaired endothelial function [49,50]. Therefore, the association of pre-pregnancy overweight or obesity and excessive GWG with the risk of PE and its phenotypes may have a common pathogenic mechanism. Additionally, studies in healthy non-pregnant volunteers have shown that weight gain is manifested primarily by visceral adipose tissue deposition rather than subcutaneous adipose tissue deposition, which not only contributes to increased risk of hypertension [51], but also results in impaired endothelial function, even in the absence of changes in blood pressure [52]. Therefore, fat distribution is also critical to the risk of hypertension. Because pre-pregnancy underweight women have a higher percentage of newly acquired visceral adipose tissue than obese women, women with excessive GWG had a higher risk of PE in pre-pregnancy underweight than in women with normal weight or overweight/obesity.

Meanwhile, we also found that inadequate GWG was a stronger risk factor for PE in pregnant women who were underweight before pregnancy compared with pre-pregnancy normal-weight women or overweight/obese women. However, the mechanism behind this finding remains unclear. Previous studies have shown that optimal maternal nutrition plays an important role in placental growth and development and that any alteration in maternal nutrient uptake or metabolism can lead to altered placental development [53,54,55]. GWG is a normal part of a healthy pregnancy and is necessary for healthy fetal development [14]. Inadequate GWG was an important manifestation of maternal malnutrition. Maternal malnutrition during pregnancy may lead to increased maternal oxidative stress through placental telomere wear, which in turn causes endothelial dysfunction and vascular injury, leading to the development of PE [56]. Among inadequate GWG group, women who are underweight before pregnancy may be at higher risk for malnutrition than women who are normal weight or overweight/obese before pregnancy.

### 4.3. Strengths and Limitations

The present study has several strengths. Firstly, this study explored the association of maternal pre-pregnancy BMI and GWG with the risk of PE and its subtypes based on a large-sample, prospective cohort design. Secondly, convenient and effective methods of communication between researchers and participants were established, including telephone calls, WeChat, and short messages, which contributed to the reduction in the rate of loss to follow-up. Furthermore, we implemented IPW to correct for potential bias. However, the study also has several limitations. Firstly, participants from this study were recruited from a single center. The results of the study might not reflect those of other institutions across the country, limiting the generalizability of the results. Secondly, data on pre-pregnancy weight and height were self-reported, thus recall bias is consequently unavoidable. Last but not least, considering that the classification criteria for obesity in the world were not completely uniform. Furthermore, the percentage of body fat differs across different ethnic populations and consequently highlights the restriction to measure obesity by BMI alone during pregnancy. Whether the results can be generalized to other ethnic groups needs further investigation due to this study was conducted on Chinese participants.

## 5. Conclusions

In conclusion, our study strongly suggests that pre-pregnancy overweight (including obesity) and abnormal GWG (including inadequate GWG and excessive GWG) are independently correlated with a higher risk of PE and that the potential risk may vary according to PE phenotypes. Moreover, the synergistic effect that may exist between pre-pregnancy BMI and GWG. Consequently, pre-pregnancy overweight and obesity should be controlled during the pre-gestation period. In addition, properly controlling the magnitude of GWG during pregnancy is also definitely necessary. However, the limitations of our study still should be carefully considered. Whether the study findings can be applied to other populations remains to be explored in future studies, especially the findings of different types of GWG with specific PE phenotypes.

## Figures and Tables

**Table 1:** The distribution of baseline characteristics of the study population.

Baseline Characteristics	Non-PE (N = 31743)	PE (N = 788)	χ^2^	*p*-Value	Maximum Standardization Difference between Groups	
	N (%)	N (%)			Before IPW	After IPW
Maternal age			81.166	<0.001	0.303	0.043
<25	1644 (5.2)	44 (5.6)				
25–29	23178 (73.0)	469 (59.5)				
30–34	5491 (17.3)	226 (28.7)				
≥35	1430 (4.5)	49 (6.2)				
Educational level			188.995	<0.001	0.457	0.041
Junior high school or below	2273 (7.2)	136 (17.3)				
Senior middle school	8907 (28.0)	305 (38.7)				
College	14668 (46.2)	257 (32.6)				
Master or above	5895 (18.6)	90 (11.4)				
Income (RMB)			6.303	0.012	0.093	0.047
≤5000	22419 (70.6)	589 (74.7)				
>5000	9324 (29.4)	199 (25.3)				
Residence			52.720	<0.001	0.258	0.014
Urban	19709 (62.1)	389 (49.4)				
Rural	12034 (37.9)	399 (50.6)				
Parity			1.417	0.234	0.043	0.045
Primiparous	15344 (48.3)	364 (46.2)				
Multiparous	16399 (51.7)	424 (53.8)				
Family history of hypertension			121.309	<0.001	0.276	0.019
No	30986 (97.6)	720 (91.4)				
Yes	757 (2.4)	68 (8.6)				
Active smoking in early pregnancy			0.219	0.640	0.016	0.006
No	31321 (98.7)	776 (98.5)				
Yes	422 (1.3)	12 (1.5)				
Passive smoking in early pregnancy			0.288	0.592	0.019	0.026
No	29562 (93.1)	730 (92.6)				
Yes	2181 (6.9)	58 (7.4)				
Drinking in early pregnancy			6.521	0.011	0.079	0.011
No	31288 (98.6)	768 (97.5)				
Yes	455 (1.4)	20 (2.5)				
Gestational diabetes mellitus			4.659	0.031	0.102	0.075
No	26762 (84.3)	642 (81.5)				
Yes	4981 (15.7)	146 (18.5)				

Note: PE = Preeclampsia; IPW = Inverse probability weighting.

**Table 2:** Associations of pre-pregnancy BMI, total GWG with the risk of preeclampsia.

Exposure	Non-PE (N = 31743)	PE (N = 788)	Model 1	*p*-Value	Model 2	*p*-Value	Model 3	*p*-Value
Pre-pregnancy BMI								
Underweight	4646 (14.6)	64 (8.1)	0.80 (0.61–1.04)	0.092	0.84 (0.65–1.10)	0.212	0.84 (0.81–0.88)	<0.001
Normal weight	22496 (70.9)	390 (49.5)	1.00					
Overweight	3881 (12.2)	215 (27.3)	3.08 (2.61–3.64)	<0.001	2.71 (2.29–3.21)	<0.001	1.62 (1.57–1.66)	<0.001
Obesity	720 (2.3)	119 (15.1)	8.32 (6.78–10.22)	<0.001	7.17 (5.82–8.84)	<0.001	2.04 (1.97–2.11)	<0.001
GWG								
Inadequate	8936 (28.2)	210 (26.6)	1.48 (1.23–1.79)	<0.001	1.44 (1.19–1.74)	<0.001	1.15 (1.12–1.19)	<0.001
Adequate	14199 (44.7)	223 (28.3)	1.00				1.00	
Excessive	8608 (27.1)	355 (45.1)	2.56 (2.17–3.03)	<0.001	2.56 (2.16–3.03)	<0.001	1.56 (1.52–1.60)	<0.001

Note: PE = Preeclampsia; BMI = Body Mass Index; GWG = Gestational weight gain. Model 1 was a crude model without any variable adjusted. Model 2 adjusted for maternal age, educational level, income (RMB), residence, family history of hypertension, drinking in early pregnancy, gestational diabetes mellitus. Model 3 adjusted for inverse probability weighting (IPW).

**Table 3:** Associations of pre-pregnancy BMI, total GWG with the risk of PE phenotypes.

Phenotypes	Exposure	Non-PE (N = 31743)	PE (N = 788)	Model 1	*p*-Value	Model 2	*p*-Value	Model 3	*p*-Value
MPE	Pre-pregnancy BMI								
	Underweight	4646 (14.6)	26 (7.3)	0.71 (0.47–1.07)	0.101	0.77 (0.51–1.16)	0.214	0.81 (0.76–0.86)	0.016
	Normal weight	22496 (70.9)	178 (50.0)	1.00					
	Overweight	3881 (12.2)	99 (27.8)	3.17 (2.48–4.05)	<0.001	2.71 (2.11–3.47)	<0.001	1.88 (1.81–1.96)	<0.001
	Obesity	720 (2.3)	53 (14.9)	8.73 (6.43–11.87)	<0.001	7.61 (5.57–10.39)	<0.001	2.93 (2.78–3.07)	<0.001
	GWG								
	Inadequate	8936 (28.2)	90 (25.3)	1.18 (0.90–1.55)	0.234	1.14 (0.87–1.50)	0.339	0.96 (0.92–1.00)	0.069
	Adequate	14199 (44.7)	121 (34.0)	1.00				1.00	
	Excessive	8608 (27.1)	145 (40.7)	1.96 (1.54–2.50)	<0.001	1.99 (1.56–2.54)	<0.001	1.51 (1.45–1.57)	<0.001
SPE	Pre-pregnancy BMI								
	Underweight	4571 (14.4)	38 (8.8)	0.87 (0.62–1.23)	0.428	0.91 (0.64–1.29)	0.584	0.82 (0.78–0.87)	<0.001
	Normal weight	22496 (70.9)	212 (49.1)	1.00					
	Overweight	3881 (12.2)	116 (26.9)	3.10 (2.48–3.90)	<0.001	2.83 (2.25–3.56)	<0.001	1.85 (1.79–1.92)	<0.001
	Obesity	720 (2.3)	66 (15.3)	8.99 (6.82–11.85)	<0.001	8.16 (6.16–10.79)	<0.001	2.67 (2.56–2.79)	<0.001
	GWG								
	Inadequate	8936 (28.2)	120 (27.8)	1.86 (1.43–2.42)	<0.001	1.82 (1.394–2.37)	<0.001	1.48 (1.42–1.54)	<0.001
	Adequate	14199 (44.7)	102 (23.6)	1.00				1.00	
	Excessive	8608 (27.1)	210 (48.6)	3.34 (2.64–4.23)	<0.001	3.34 (2.64- 4.23)	<0.001	2.12 (2.04–2.19)	<0.001
EOPE	Pre-pregnancy BMI								
	Underweight	4646 (14.6)	31 (9.4)	0.97 (0.66–1.43)	0.897	1.08 (0.73–1.59)	0.700	0.97 (0.92–1.03)	0.351
	Normal weight	22496 (70.9)	154 (46.5)	1.00				1.00	
	Overweight	3881 (12.2)	105 (31.7)	3.87 (3.02–4.96)	<0.001	3.36 (2.61–4.32)	<0.001	2.37 (2.28–2.46)	<0.001
	Obesity	720 (2.3)	41 (12.4)	7.92 (5.62–11.18)	<0.001	7.08 (4.99–10.03)	<0.001	3.06 (2.90–3.24)	<0.001
	GWG								
	Inadequate	8936 (28.2)	91 (27.5)	1.67 (1.25–2.25)	0.001	1.63 (1.21–2.19)	0.001	1.39 (1.33–1.46)	<0.001
	Adequate	14199 (44.7)	86 (26.0)	1.00				1.00	
	Excessive	8608 (27.1)	154 (46.5)	2.92 (2.24–3.80)	<0.001	2.94 (2.26–3.83)	<0.001	2.13 (2.04–2.21)	<0.001
LOPE	Pre-pregnancy BMI								
	Underweight	4646 (14.6)	33 (7.2)	0.68 (0.47–0.98)	0.037	0.70 (0.48–1.01)	0.057	0.72 (0.68–0.76)	<0.001
	Normal weight	22496 (70.9)	236 (51.6)	1.00					
	Overweight	3881 (12.2)	110 (24.1)	2.65 (2.12–3.33)	<0.001	2.38 (1.90–3.00)	<0.001	1.62 (1.56–1.68)	<0.001
	Obesity	720 (2.3)	78 (17.1)	9.42 (7.29–12.16)	<0.001	8.40 (6.47–10.90)	<0.001	2.53 (2.43–2.63)	<0.001
	GWG								
	Inadequate	8936 (28.2)	119 (26.0)	1.37 (1.07–1.76)	0.011	1.34 (1.05–1.71)	0.020	1.09 (1.05–1.14)	<0.001
	Adequate	14199 (44.7)	137 (30.0)	1.00				1.00	
	Excessive	8608 (27.1)	201 (44.0)	2.38 (1.92–2.97)	<0.001	2.40 (1.93–2.98)	<0.001	1.64 (1.58–1.69)	<0.001

Note: BMI = Body Mass Index; GWG = Gestational weight gain; PE = Preeclampsia; MPE = Mild Preeclampsia; SPE = Severe Preeclampsia; EOPE = Early-onset Preeclampsia; LOPE = Late-onset Preeclampsia; Model 1 was a crude model without any variable adjusted; Model 2 adjusted for maternal age, educational level, income (RMB), residence, family history of hypertension, drinking in early pregnancy, gestational diabetes mellitus; Model 3 adjusted for inverse probability weighting (IPW).

**Table 4:** Stratified analysis of association between GWG and the risk of PE and its phenotypes based on different pre-pregnancy BMI level.

Phenotypes		Non-PE	PE	Incidence % [95%CI)	RR (95%CI) *	*p*-Value
PE	Underweight					
	Inadequate	1533 (33.0)	30 (46.9)	1.92 (1.35–2.73)	2.43 (2.21–2.67)	<0.001
	Adequate	2313 (49.8)	14 (21.8)	0.60 (0.36–1.01)		
	Excessive	800 (17.2)	20 (31.3)	2.44 (1.59–3.74)	2.00 (1.78–2.25)	<0.001
	Normal weight					
	Inadequate	6887 (30.6)	134 (34.4)	1.91 (1.62–2.26)	1.25 (1.20–1.30)	<0.001
	Adequate	10242 (45.5)	117 (30.0)	1.13 (0.94–1.35)		
	Excessive	5367 (23.9)	139 (35.6)	2.52 (2.14–2.97)	1.63 (1.56–1.69)	<0.001
	Overweight/Obesity					
	Inadequate	516 (11.2)	46 (13.8)	8.19 (6.20–10.75)	1.02 (0.95–1.10)	0.620
	Adequate	1644 (35.7)	92 (27.5)	5.30 (4.34–6.46)		
	Excessive	2441 (53.1)	196 (58.7)	7.43 (6.49–8.49)	1.08 (1.04–1.13)	<0.001
MPE	Underweight					
	Inadequate	1533 (33.0)	14 (53.8)	0.90 (0.54–1.51)	6.04 (5.01–7.27)	<0.001
	Adequate	2313 (49.8)	3 (11.5)	0.13 (0.04–0.38)		
	Excessive	800 (17.2)	9 (34.6)	1.11 (0.58–2.10)	5.59 (4.57–6.85)	<0.001
	Normal weight					
	Inadequate	6887 (30.6)	55 (30.9)	0.79 (0.61–1.03)	0.86 (0.81–0.91)	<0.001
	Adequate	10242 (45.5)	68 (38.2)	0.66 (0.52–0.84)		
	Excessive	5367 (23.9)	55 (30.9)	1.01 (0.78–1.31)	1.37 (1.30–1.44)	<0.001
	Overweight/Obesity					
	Inadequate	516 (11.2)	21 (13.8)	3.91 (2.57–5.90)	0.96 (0.84–1.09)	0.496
	Adequate	1644 (35.7)	50 (32.9)	2.95 (2.24–3.87)		
	Excessive	2441 (53.1)	81 (53.3)	3.21 (2.59–3.97)	1.23 (1.14–1.32)	<0.001
SPE	Underweight					
	Inadequate	1533 (33.0)	16 (42.2)	1.03 (0.63–1.67)	2.03 (1.81–2.28)	<0.001
	Adequate	2313 (49.8)	11 (28.9)	0.47 (0.26–0.84)		
	Excessive	800 (17.2)	11 (28.9)	1.36 (0.76–2.42)	1.27 (1.08–1.50)	0.003
	Normal weight					
	Inadequate	6887 (30.6)	79 (37.3)	1.13 (0.91–1.41)	1.92 (1.81–2.03)	<0.001
	Adequate	10242 (45.5)	49 (23.1)	0.48 (0.36–0.63)		
	Excessive	5367 (23.9)	84 (39.6)	1.54 (1.25–1.90)	2.51 (2.38–2.65)	<0.001
	Overweight/Obesity					
	Inadequate	516 (11.2)	25 (13.7)	4.62 (3.15–6.73)	1.14 (1.05–1.23)	0.002
	Adequate	1644 (35.7)	42 (23.1)	2.49 (1.85–3.35)		
	Excessive	2441 (53.1)	115 (63.2)	4.50 (3.76–5.37)	1.22 (1.15–1.29)	<0.001
EOPE	Underweight					
	Inadequate	1533 (33.0)	13 (41.9)	0.84 (0.49–1.43)	3.40 (2.93–3.96)	<0.001
	Adequate	2313 (49.8)	6 (19.4)	0.26 (0.12–0.57)		
	Excessive	800 (17.2)	12 (38.7)	1.48 (0.85–2.57)	3.67 (3.12–4.33)	<0.001
	Normal weight					
	Inadequate	6887 (30.6)	59 (38.3)	0.85 (0.66–1.09)	1.59 (1.49–1.69)	<0.001
	Adequate	10242 (45.5)	44 (28.6)	0.43 (0.32–0.58)		
	Excessive	5367 (23.9)	51 (33.1)	0.94 (0.72–1.23)	1.88 (1.76–2.00)	<0.001
	Overweight/Obesity					
	Inadequate	516 (11.2)	19 (13.0)	3.55 (2.28–5.48)	1.07 (0.97–1.19)	0.171
	Adequate	1644 (35.7)	36 (24.7)	2.14 (1.55–2.95)		
	Excessive	2441 (53.1)	91 (62.3)	3.59 (2.93–4.39)	1.30 (1.22–1.38)	<0.001
LOPE	Underweight					
	Inadequate	1533 (33.0)	17 (51.6)	1.10 (0.69–1.75)	2.59 (2.29–2.93)	<0.001
	Adequate	2313 (49.8)	8 (24.2)	0.34 (0.17–0.67)		
	Excessive	800 (17.2)	8 (24.2)	0.99 (0.05–1.94)	1.27 (1.06–1.52)	0.009
	Normal weight					
	Inadequate	6887 (30.6)	75 (31.8)	1.08 (0.86–1.35)	1.17 (1.11–1.23)	<0.001
	Adequate	10242 (45.5)	73 (30.9)	0.71 (0.57–0.89)		
	Excessive	5367 (23.9)	88 (37.3)	1.61 (1.31–1.98)	1.81 (1.73–1.90)	<0.001
	Overweight/Obesity					
	Inadequate	516 (11.2)	27 (14.4)	4.97 (3.44–7.14)	1.00 (0.92–1.09)	0.977
	Adequate	1644 (35.7)	56 (29.7)	3.29 (2.54–4.25)		
	Excessive	2441 (53.1)	105 (55.9)	4.12 (3.41–4.96)	1.03 (0.98–1.09)	0.196

Note: BMI = Body Mass Index; GWG = Gestational weight gain; PE = Preeclampsia; MPE = Mild Preeclampsia; SPE = Severe Preeclampsia; EOPE = Early-onset Preeclampsia; LOPE = Late-onset Preeclampsia; * adjusted for inverse probability weighting (IPW). *p*-value for interaction: 0.233, 0.904, 0.125, 0.153 and 0.731, respectively.

## Data Availability

The data presented in this study are available on request from the corresponding author.
